# SOM3355: a unique pharmacological profile combining VMAT1 inhibition, VMAT2-mediated dopamine modulation, and β_1_-adrenergic antagonism for the treatment of movement and neuropsychiatric disorders

**DOI:** 10.3389/fphar.2026.1824708

**Published:** 2026-05-19

**Authors:** Emily S. Turilli-Ghisolfi, Svein Isungset Støve, Oscar Huertas, Trond-André Kråkenes, Knut Teigen, Silvia Panigone, Rossella Medori

**Affiliations:** 1 SOM Innovation Biotech SA, Barcelona, Spain; 2 Department of Biomedicine, University of Bergen, Bergen, Norway; 3 Department of Neurology, Neuro-SysMed, Haukeland University Hospital, Bergen, Norway

**Keywords:** beta-blockers, bevantolol, dopamine, Huntington’s disease, SOM3355, tardive dyskinesia, VMAT

## Abstract

Unmet needs in hyperkinetic movement disorders (HMDs) within neuropsychiatric conditions include better treatments for HMDs in complex disorders like Huntington’s disease (HD), Tourette syndrome and psychiatric comorbidities like tardive dyskinesia. The symptomatology of HD comprises chorea, a disabling movement disorder, neuropsychiatric disturbances and cognitive decline. VMAT2 inhibitors like tetrabenazine and its derivatives, as well as antipsychotics, are used for the treatment of HMDs but they may worsen motor function or the underlying neuropsychiatric symptoms. Specifically targeted therapies improving management of motor and non-motor symptoms with a safety profile suited for continued chronic treatment are needed. We describe here the novel mechanism of action of SOM3355, a CNS-permeant selective β_1_-blocker (bevantolol) found to have additional VMAT2 and VMAT1 modulation activity. SOM3355 is a clinical drug candidate for the treatment of HD that has shown positive clinical effects in the motor-behavioral/psychiatric-cognitive symptom triad characteristic of the disease. *In vitro* and *in vivo* studies were conducted to evaluate SOM3355’s inhibitory activity at VMAT1, VMAT2 and CNS targets. Functional uptake and radioligand binding assays were performed in rat vesicles and human recombinant systems. SOM3355 inhibited VMAT2-mediated dopamine uptake comparably to tetrabenazine but displayed markedly lower affinity for the α-dihydrotetrabenazine binding site, suggesting a distinct interaction mode. Unlike tetrabenazine, SOM3355 potently inhibited VMAT1, a vesicular monoamine transporter present in different brain areas and implicated in neuropsychiatric regulation. Additionally, SOM3355 exhibited negligible off-target CNS binding, consistent with the favorable CNS safety profile observed in clinical trials. By combining synergistic β_1_-adrenergic antagonism, VMAT2-mediated dopamine modulation, and VMAT1 inhibition, SOM3355 may address monoamine neurotransmitter imbalances within basal ganglia and cortical circuits involved in motor and neuropsychiatric manifestations of HD. In summary, the translational profile of SOM3355, aligned with its clinical evaluation to date, supports its positioning as a potential novel therapeutic alternative for the treatment of HMDs and neuropsychiatric conditions like HD, tardive dyskinesia and Tourette Syndrome.

## Graphical Abstract

**Figure:**
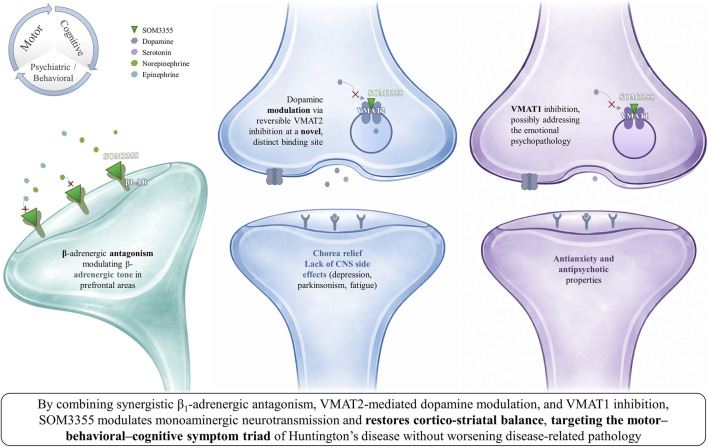

## 1 Introduction

Movement is one of the robust bridges between neurology and psychiatry according to the biggest expert Charles David Marsden (Marsden, 1989). This is demonstrated by the frequency of movement disorders in psychiatric and neurological diseases, stemming from medication side effects (like tardive dyskinesia and drug-induced parkinsonism), the diseases themselves (e.g., chorea in Huntington’s Disease (HD), tics in Tourette Syndrome), or underlying neurological dysfunction. Movement disorders include excessive or insufficient movement affecting a patient’s function, social life and ultimately quality of life.

**FIGURE 1:**
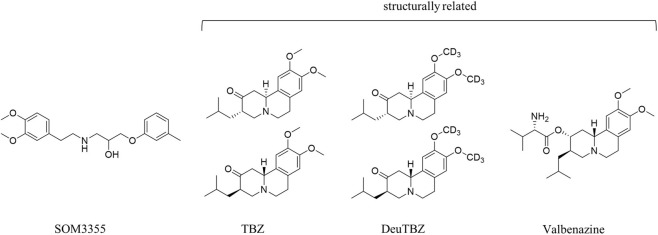
Chemical structure of SOM3355, compared with TBZ and its derivatives deuTBZ and valbenazine.

HD is a progressive, autosomal dominant neurodegenerative disorder characterized by a triad of motor dysfunction, cognitive decline and psychiatric disturbances (Adam and Jankovic, 2008). The hallmark motor symptom of HD is chorea, an involuntary, irregular movement disorder that significantly impairs quality of life and functional independence. As the disease progresses, patients may also develop dystonia, rigidity, bradykinesia and akathisia, further complicating management and care (Bachoud-Lévi et al., 2019). Psychiatric symptoms vary widely among patients. Depression, anxiety, agitation and aggression are common, but apathy, perseverations, hallucinations and delusions may appear as well, making HD a complex multisymptomatic disorder including increasing cognitive decline–manifested as memory loss, poor judgment and impaired concentration (Peavy et al., 2010). Despite decades of research, no curative therapies exist for movement disorders in psychiatry and neurology. The current HD-specific authorized treatments are solely aiming to control chorea and belong to the class of vesicular monoamine transporter type 2 (VMAT2) inhibitors. Tetrabenazine (TBZ) and its two derivatives deutetrabenazine (deuTBZ) and valbenazine (Figure 1) are effective on chorea, but have a side-effect profile that includes depression, cognitive decline, parkinsonism, dysphagia, sedation/somnolence and akathisia (Frank et al., 2022; U.S. Food and Drug Administration and Center for Drug Evaluation and Research, 2017a; U.S. Food and Drug Administration and Center for Drug Evaluation and Research, 2017b; U.S. Food and Drug Administration and Center for Drug Evaluation and Research, 2023), thus potentially exacerbating the intrinsic psychiatric symptoms of HD and blurring the distinction between drug-induced adverse events and the progression of the underlying disease (XENAZINE tetrabenazine tablets and for oral use, 2025). Tetrabenazine is associated with high real-world discontinuation rates, largely driven by tolerability issues and concerns regarding neuropsychiatric adverse effects. Consequently, a substantial unmet need remains for safe long-term therapies targeting both motor and neuropsychiatric symptoms (Sung et al., 2018; Claassen et al., 2022).

SOM3355 (bevantolol hydrochloride, Figure 1), originally developed by Parke-Davis (Pfizer Inc.) as a β_1_-blocker and later licensed to Nippon Chemiphar for development in Asia, has been used clinically since the 1980s for the treatment of hypertension and angina pectoris, with an established safety profile and no evidence of CNS liabilities. Preclinical studies have demonstrated its ability to penetrate the CNS and to modulate monoaminergic signaling in presynaptic neurons, together with β-adrenergic antagonism.

SOM3355 is currently under investigation for the treatment of HD. In a proof-of-concept study, SOM3355 administered at 400 mg/day demonstrated a significant improvement in chorea (UHDRS-TMC score) compared to placebo, with no worsening of depression and suicidality in patients not excluded based on these symptoms at baseline. In a recent randomized, double-blind, placebo-controlled phase 2b trial testing 400 mg/day and 600 mg/day, alongside statistically significant improvements in chorea, SOM3355 showed a positive impact on behavioral symptoms that were present at baseline (anxiety, apathy, compulsive, disruptive and irritable behaviors, and perseverative thinking), as well as a progressive improvement in depressive symptoms, with no observations of suicidality or suicide attempts in association with SOM3355, but two cases on placebo (SOM Innovation Biotech SA, 2025).

Despite previous concerns with psychiatric adverse events during β-blocker use (Verbeek et al., 2011; Bornand et al., 2022; Boal et al., 2016), a growing body of clinical data support the use of CNS-permeant β-blockers in neuropsychiatric symptomatology associated with a variety of conditions, with reported efficacy in anxiety, aggression, irritability, working memory, tremor and akathisia (Liu et al., 2017; Wu et al., 2019; Sagar-Ouriaghli et al., 2018; Brouwers et al., 2016; Clay et al., 2019; Kessing et al., 2020; Kim et al., 2019; Battes et al., 2012; Tofler et al., 2020).

This article presents the newly characterized non-clinical polypharmacology of SOM3355, aiming to highlight its potential key role within the therapeutic landscape of HD and explore its potential advantages over existing available treatments.

## 2 Results

### 2.1 SOM3355 shows monoaminergic modulation via VMAT2 with a distinct interaction profile compared with TBZ

**FIGURE 2:**
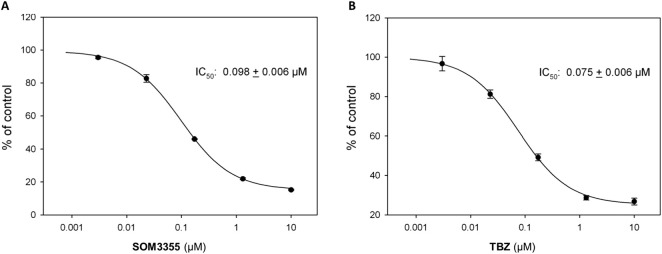
Dose-response curve of **(A)** SOM3355 and **(B)** TBZ for VMAT2-mediated [^3^H]-DA uptake into rat striatal vesicles.

**FIGURE 3:**
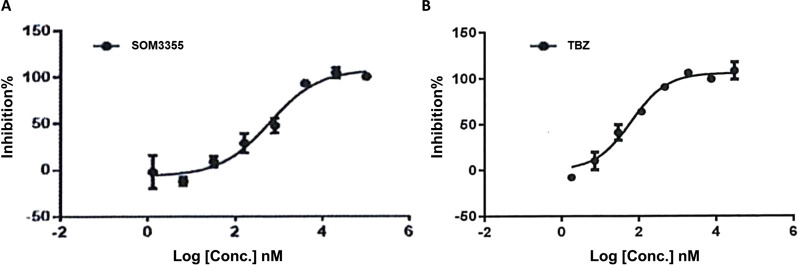
Dose-response curve of **(A)** SOM3355 and **(B)** TBZ for VMAT2-mediated [^3^H]-5-HT uptake into human vesicles from transfected HEK293 cells.

**FIGURE 4:**
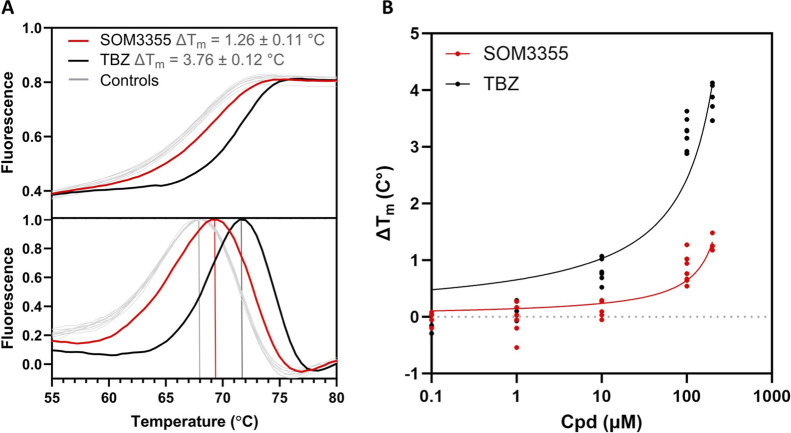
VMAT2 stabilization assessment by DSF. **(A)** DSF-monitored comparative unfolding of rat VMAT2. Representative unfolding curves (upper panel) and first derivative (lower panel), providing the apparent melting temperature (T_m_) values for VMAT2 alone (DMSO control; gray lines) or in the presence of 200 µM SOM3355 (red lines) or TBZ (black lines). The T_m_ value for the control was 67.9 °C ± 0.21 °C, resulting in the average protein melting temperature shift (ΔT_m_) values indicated in the figure. **(B)** Concentration dependent DSF comparison with 0.1, 1, 10, 100 and 200 μM SOM3355 vs TBZ.

In an exploratory *in vitro* screening, testing 23 β-blocking compounds at a single concentration of 10 µM in triplicate for the inhibition of VMAT2-mediated [^3^H]-DA uptake into rat striatal vesicles, SOM3355 emerged among the active compounds (Supplementary Table S1). Upon dose-response investigation in the same system, SOM3355 was shown to modulate VMAT2-mediated dopamine uptake with an IC_50_ of 98 nM, while TBZ inhibited VMAT2 with an IC_50_ of 75 nM (Figure 2). The IC_50_ ratio between SOM3355 and TBZ was confirmed in further tests in rat cortical vesicles (Supplementary Figure S1), as well as in vesicles from human VMAT2-transfected HEK293 cells (Supplementary Figure S2; Table 1). Of note, rat VMAT2 shares more than 91% sequence identity with human VMAT2. In the same human cellular system, SOM3355 inhibited [^3^H]-5-HT uptake with an IC_50_ of 600.5 nM (Figure 3), exhibiting a potency more than 10-fold lower than that of TBZ (IC_50_ = 63.18 nM), indicating a substrate-dependent difference in inhibitory strength (Table 1). In a differential scanning fluorimetry (DSF) assessment (Figure 4), SOM3355 stabilized purified rat VMAT2 with a ΔT_m_ of 1.26 °C ± 0.11 °C, presenting a milder stabilization effect than TBZ (ΔT_m_ = 3.76 °C ± 0.27 °C). In radioligand binding assays in isolated vesicles from human VMAT2-transfected HEK293 cells, SOM3355 displaced [^3^H]-α-dihydrotetrabenazine ([^3^H]-α-DHTBZ), the active metabolite of TBZ, with an IC_50_ of 1.35 µM, compared with 19.23 nM for TBZ, demonstrating a lower affinity of SOM3355 for the α-DHTBZ binding site (Table 2; Supplementary Figure S3). The strong difference between SOM3355 and TBZ in [^3^H]-α-DHTBZ displacement was confirmed in a further assay in rat brain membranes (Supplementary Figure S4). In [^3^H]-reserpine binding assays in VMAT2-transfected HEK293 isolated vesicles, SOM3355 showed an IC_50_ of 267 nM versus 32.4 nM for TBZ, while reserpine itself remained the most potent compound (IC_50_ = 1.32 nM).

**TABLE 1:** Comparative inhibition of human VMAT2-mediated uptake of monoamine neurotransmitters in transfected HEK293 cells. [^3^H]-α-DHTBZ = tritiated alpha-dihydrotetrabenazine; [^3^H]-5-HT = tritiated serotonin; [^3^H]-DA = tritiated dopamine; ND = Not Determined.

​	IC_50_ (nM)	
Compound	[^3^H]-DA uptake	[^3^H]-5-HT uptake
SOM3355	65.64	600.5
TBZ	31.69	63.18
Reserpine	0.74	0.47
DA	5470	ND
5-HT	ND	2105

**TABLE 2:** Human VMAT2 comparative radioligand binding in transfected HEK293 cells. [^3^H]-α-DHTBZ = tritiated alpha-dihydrotetrabenazine; ND = Not Determined.

​	IC_50_ (nM)	
Compound	[^3^H]-α-DHTBZ binding	[^3^H]-reserpine binding
SOM3355	1353	267
TBZ	19.23	32.40
Reserpine	ND	1.32

### 2.2 SOM3355 is a VMAT1 inhibitor

**FIGURE 5:**
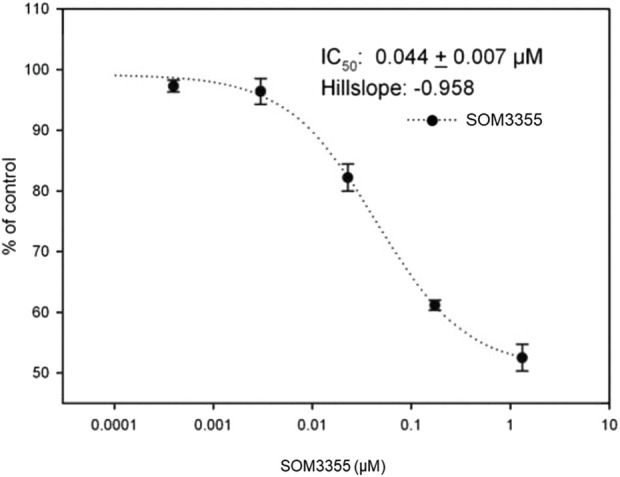
SOM3355 dose-response curve for [^3^H]-5-HT uptake into rat VMAT1-expressing membrane vesicles from transfected CHO cells.

In an exploratory comparative screening for inhibition of initial rates of [^3^H]-5-HT uptake into rat VMAT1-expressing membrane vesicles from transfected CHO cells at 10 μM, SOM3355 emerged as a very active compound, while TBZ and its metabolite trans-(2,3)-DHTBZ showed negligible to no activity (Supplementary Table S2). In a subsequent dose-response investigation in the same system, SOM3355 potently inhibited VMAT1-mediated uptake of serotonin with an IC_50_ of 44 nM (Figure 5).

### 2.3 Comparative CNS target profiling identifies a clean, favorable non-dopaminergic binding signature for SOM3355 over TBZ and α-DHTBZ

To elucidate the pharmacological selectivity of SOM3355 in the CNS and evaluate potential off-target effects, a comparative radioligand binding screening against a panel of 32 CNS targets (human G-protein coupled receptors, human monoamine transporters, ion channels and selected enzymes) was conducted for SOM3355, TBZ and α-DHTBZ at a single, high concentration of 10 µM (Table 3; Supplementary Figure S5). SOM3355 showed a clean, different activity profile compared with TBZ and α-DHTBZ.

**TABLE 3:** Comparative CNS target panel profiling results for SOM3355, TBZ and α-DHTBZ tested at 10 µM (% inhibition of control specific binding). α_1A_, α_2C_ = adrenergic receptor subtypes 1A and 2C; D_2s_ = dopamine D_2_ receptor (short isoform); D_3_ = dopamine D_3_ receptor; D_4.4_ = dopamine D_4_ receptor; MT_3_ (ML_2_) = melatonin receptor type 3; 5-HT = 5-hydroxytryptamine (serotonin) receptor subtypes (1A, 2A, 2C); Sigma = sigma receptor (non-selective); Na^+^ Channel = voltage-gated sodium channel; NET = norepinephrine transporter; DAT = dopamine transporter; 5-HT T = serotonin transporter.

Target	SOM3355	TBZ	α-DHTBZ
α_1A_ adrenergic (Antagonist radioligand)	85.8	8.7	9.7
α_2C_ adrenergic(Antagonist radioligand)	41.5	50.8	59.4
D_2s_ (Agonist radioligand)	46.2	89.3	89.6
D_3_ (Antagonist radioligand)	38.2	69.0	84.3
D_4.4_ (Antagonist radioligand)	48.5	79.5	39.9
MT_3_ (ML_2_) (Agonist radioligand)	−3.5	77.3	−1.1
5-HT_1A_ (Agonist radioligand)	95.0	87.3	90.4
5-HT_2A_ (Agonist radioligand)	57.4	31.8	19.3
5-HT_2C_ (Agonist radioligand)	81.7	57.6	23.2
Sigma (non selective) (Agonist radioligand)	62.5	71.1	93.3
Na^+^ channel (site 2) (Antagonist radioligand)	64.5	11.2	14.6
NET (Antagonist radioligand)	79.1	14.4	−2.6
DAT (Antagonist radioligand)	53.4	2.2	6.3
5-HT T (Antagonist radioligand)	48.4	6.0	18.6

Regarding dopaminergic targets, SOM3355 exhibited negligible affinity (<50% inhibition of control specific binding) for D_2s_, D_3_, and D_4.4_ receptors, while TBZ and α-DHTBZ showed marked binding to these subtypes, inhibiting binding of the agonist radioligand to D_2s_ (around 90%), and antagonist radioligands to D_3_ and D_4.4_ receptors (up to 84.3%). Moreover, SOM3355 displayed affinity for 5-HT_1A_, 5-HT_2A_ and 5-HT_2C_ receptors, while no activity appeared at the serotonin transporter.

To assess whether the binding signals observed at NET (79.1%) and DAT (53.4%) were of functional relevance, dedicated uptake assays were performed. SOM3355 exhibited only weak inhibitory activity at these transporters, with IC_50_ values in the micromolar range (4.47 µM and 4.68 µM, respectively), supporting a favorable safety profile in the context of movement disorders (Supplementary Figure S6). Moreover, SOM3355 showed no significant inhibitory activity against acetylcholinesterase (13.8% inhibitory activity versus control), GABA transaminase (−8.0% inhibitory activity versus control), and tyrosine hydroxylase (−5.9% inhibitory activity versus control), with values attributable to variability of the signal around the control level (Supplementary Table S3).

### 2.4 SOM3355 is a β_1_-adrenergic antagonist

The activity of SOM3355 as a β_1_-adrenergic receptor antagonist with greater potency at β_1_ than β_2_ was confirmed in *in vitro* functional cAMP-HTRF assays, with IC_50_ values of 35 nM and 330 nM respectively (Supplementary Figure S7).

Functional antagonism at cardiac β-adrenergic receptors was further assessed in isolated guinea pig atrial preparations, where SOM3355 competitively antagonized isoproterenol-induced chronotropic and inotropic responses with a pA_2_ value of 7.2, comparable to those of metoprolol and propranolol (undisclosed data by Nippon Chemiphar Co., Ltd). In *in vivo* studies in anesthetized dogs, SOM3355 dose-dependently attenuated isoproterenol-induced increases in heart rate and blood pressure, with near-maximal antagonism observed at doses of 2–6 mg/kg (undisclosed data by Nippon Chemiphar Co., Ltd). Functional profiling of SOM3355 metabolites revealed weak residual β_1_-blocking activity across all derivatives, with one metabolite notably displaying intrinsic sympathomimetic properties. In both *ex vivo* guinea pig atrial assays and *in vivo* anaesthetized dogs, SOM3355 exhibited superior inotropic and chronotropic potency compared to its metabolites, one of which retained only 1/2000th the activity of the parent drug.

Moreover, low-to-absent α-adrenergic activity was detected. In radioligand binding studies, SOM3355 showed minimal activity at the α_1_-receptor, and no activity at the α_2_-receptor (Table 3). A follow-up dose-response evaluation determined an IC_50_ of 5.6 µM for SOM3355 at the α_1_-receptor (Supplementary Figure S8), consistent with its weak peripheral adrenergic effects in *ex vivo* cardiovascular studies (undisclosed data by Nippon Chemiphar Co., Ltd). In rabbit thoracic aorta preparations, SOM3355 antagonized the effect of norepinephrine with a pA_2_ value of 5.49 ± 0.05. Of note, *in vivo*, the potency of SOM3355 against isoproterenol in anaesthetized dogs was approximately 14-times that of antagonizing the vasopressor effects of the α_1_-stimulant phenylephrine, confirming that the potential α_1_-blocking effects of SOM3355 are much less potent than the β_1_-blocking effects.

## 3 Discussion

Huntington’s Disease represents a complex intersection of neurology and psychiatry, characterized by a debilitating triad of motor, cognitive, and psychiatric symptoms. The pharmacological management of the hyperkinetic component of this triad relies primarily on targeting VMAT2, predominantly expressed in the basal ganglia, and responsible for monoamine neurotransmitter transport. In the early HD stages, striatal DA neurotransmission is increased leading to chorea that can be alleviated by depleting DA stores (Cepeda et al., 2014; Ledonne and Mercuri, 2017). Up to now, specific treatments for chorea are represented by the VMAT2 inhibitors TBZ and its derivatives, deuTBZ and valbenazine, that predominantly act by reducing the presynaptic internalization and storage of DA into vesicles, leading to DA depletion in nerve terminals and subsequent reduced release and signaling. Despite efficacy in chorea management, the potency and selectivity of this mechanism however causes or exacerbates other motor symptoms like parkinsonism and akathisia in later stages of HD that are associated with DA deficits in the basal ganglia and hypokinesia (Paek, 2020; Canney et al., 1995; Kim, 2017), and worsens psychiatric symptoms. TBZ is associated with dose-dependent side effects, including depression with increased risk of suicidality and somnolence/sedation (Chitnis and Karunapuzha, 2009). DeuTBZ has a longer half-life compared to TBZ, with reduced peak plasma fluctuations that account for lower incidence of somnolence but still requires careful titration and monitoring for neuropsychiatric adverse events.

Consequently, a substantial unmet need emerges for safer therapies capable of controlling motor symptoms without exacerbating the patient’s motor and psychiatric burden. In this context, β-blockers offer a clinically grounded rationale, with proven efficacy in treating neuropsychiatric symptomatology and antipsychotic-induced movement disorders. β_1_-adrenergic antagonists have long been used in the management of akathisia and other movement-related disorders (Thippaiah et al., 2025), and are increasingly prescribed for treating anxiety, with propranolol having a Product License in the United Kingdom. Moreover, recent observational data have reported an association between long-term β-blocker use and delayed onset and slower progression of HD, suggesting that modulation of adrenergic signaling may be relevant to both symptomatic and potentially disease-related mechanisms (Schultz et al., 2025).

SOM3355 (bevantolol hydrochloride), identified through the proprietary SOM^AI^PRO platform, is a CNS-permeant β-blocker and is currently a clinical candidate for the treatment of HD. The activity of SOM3355 at VMATs was studied, alongside a deeper characterization of its pharmacological profile, in comparative assays versus other β-blockers and versus VMAT2 inhibitors. The five prerequisite for β-blockers to be suited for the treatment of movement disorders are i) to cross the blood-brain-barrier, to have ii) VMAT2 inhibitory activity with a potency comparable to TBZ but different binding mode with lower affinity for other monoamines like serotonin to maximize neuropsychiatric safety, iii) selectivity for β_1_-receptor to minimize unacceptable cardiovascular adverse events (associated with α_1_-and α_2_-adrenergic receptor inhibition) and respiratory adverse events like asthma (linked to β_2_-adrenergic receptor inhibition), iv) moderate β-adrenergic inhibitory activity to limit adverse events in non-hypertensive patients, given the need for doses higher than for the treatment of hypertension, and v) a favorable cytochrome P450 metabolic profile with limit propensity for drug-drug interactions in the neuropsychiatric indications. Four β-blockers with VMAT2 inhibitory activity were identified by SOM Biotech: SOM3355, labetalol, nebivolol and bucindolol, and another one, carvedilol, by researchers at the University of Bergen (Støve et al., 2022). SOM3355 was found to be the only β-blocker to satisfy all requirements necessary for the chronic medicinal treatment of movement disorders and psychiatric and neurological disturbances like HD, Tourette syndrome and tardive dyskinesia (Table 4).

**TABLE 4:** Comparative overview of β-blockers with VMAT2 inhibitory activity with respect to selection criteria for targeting movement psychiatric and behavioral disorders.

Compound	Mechanism of action (MoA)	VMAT2 IC_50_ (µM)	Met criteria	Barriers to use in neuropsychiatry
SOM3355	VMAT1/VMAT2 inhibitor and β_1_-blocker	0.098 ± 0.006	1,2,3,4,5	None
Labetalol	α- and non-selective β-blocker	0.202 ± 0.008	None: prerequisite not met	Does not cross the blood-brain-barrier
Nebivolol	Selective β_1_-blocker	0.008 ± 0.0054	1,2,3,4	Low therapeutic window (too high potency), with cardiotoxicity for doses >40 mg
Carvedilol	α- and non-selective β-blocker	0.227 ± 0.053	1,2	Lower VMAT2 inhibitory potency, same binding site as TBZ, no VMAT1 inhibition, receptor profile including α-receptor does not allow higher doses needed in neuropsychiatry
Bucindolol	α- and non-selective β-blocker	0.453 ± 0.101	1,2	Low VMAT2 inhibitor activity and effects on α-receptors do not allow use of the needed higher doses

The pharmacological evaluation of SOM3355 reveals a compound with a unique and multifaceted pharmacological profile including β_1_-adrenergic-, VMAT1-and VMAT2-inhibitory activities. This positions the compound as a promising candidate for the treatment of conditions with coexisting movement, psychiatric and neurological disorders, stemming from medication side effects (like tardive dyskinesia and drug-induced parkinsonism) or the diseases themselves (like chorea in HD and tics in Tourette syndrome).

While non-selective β-blockers or compounds with concurrent α-adrenergic activity are often restricted by cardiovascular and respiratory dose-limiting toxicities, SOM3355 avoids these liabilities through its pronounced β_1_ selectivity. The functional separation between its β_1_-blocking efficacy and its negligible α_1_ and β_2_ activities translates clinically to a reduced risk of orthostatic hypotension and bronchospasm, respectively. This autonomic safety margin allows for safe administration of the doses typically required for efficient CNS penetration and target engagement in movement disorders. In addition, the absence of significant pharmacological activity among its metabolites ensures that this therapeutic window is driven strictly by the parent compound, facilitating predictable dosing and monitoring in a vulnerable neurological population.

Furthermore, while SOM3355 effectively modulates VMAT2-mediated dopamine uptake with nanomolar potency, its interaction profile is markedly distinct from that of classical VMAT2 inhibitors like TBZ and its derivatives. Radioligand assays revealed a significant functional-binding dissociation that is consistent with a different interaction mode at VMAT2 compared with TBZ: SOM3355 displaces α-DHTBZ, the main active metabolite of TBZ, with an approximately 70-fold lower affinity than TBZ, and reserpine with an 8-fold lower affinity than TBZ. Furthermore, DSF measurements revealed that SOM3355 induces a milder thermal stabilization of VMAT2 (ΔT_m_ of 1.26 °C ± 0.11 °C) compared with that of TBZ (ΔT_m_ = 3.76 °C ± 0.27 °C). As described in the literature for DSF testing of VMAT2-inhibiting compounds, smaller ΔT_m_ shifts are generally consistent with a milder and reversible interaction (Støve et al., 2022).

It is reported that TBZ binds VMAT2 in a lumen-facing conformation, locking the luminal gating lid in a rigid, “dead-end” occluded state to arrest the transport cycle (Scherman and Henry, 1983; Ugolev et al., 2013; Chen et al., 2013; Dalton et al., 2024; Kaur et al., 2016; Yaffe et al., 2016). The binding site of TBZ only partially overlaps with that of reserpine, which binds in a cytoplasm-facing conformation, expanding the vestibule and blocking substrate access. By contrast, our data strongly suggest that SOM3355 interacts with VMAT2 via a different binding mode than classical VMAT2 inhibitors, and potentially at a different binding site from that of TBZ. Moreover, SOM3355 exhibited a 10-fold less potency for VMAT2-mediated serotonin uptake compared with TBZ, highlighting a substrate-dependent difference in inhibitory strength that may be consistent with a different binding site and with SOM3355’s more favorable safety profile observed in clinical studies.

Moreover, SOM3355 was found to potently inhibit VMAT1 with an IC_50_ of 44 nM, further differentiating its mechanism of action from TBZ and its derivatives, which selectively target VMAT2 with no activity on VMAT1. VMAT1, another subtype of the vesicular neurotransmitter transporter, differs from VMAT2 in the expression patterns within the CNS and the peripheral nervous system. While VMAT2 is located primarily in the basal ganglia, VMAT1 is located in the substantia nigra, hippocampus, thalamus, amygdala and frontal lobe. Indeed, modulation of VMAT1 carries clinical relevance (Ranjbar-Slamloo and Fazlali, 2020; Clark and Noudoost, 2014; Aono et al., 2007): functional effects of VMAT1 overactivity are reported to be associated with several phenotypes in schizophrenia and anxiety-related personality traits, and notably, certain antipsychotic drugs like ziprasidone inhibit VMAT1 in addition to VMAT2. In the context of HD, the inhibition of VMAT1 might correct neurotransmitter imbalances–such as the relative “hyperfunction” in prefrontal cortex (reported to be relatively decreased) and in the amygdala (reported to be relatively increased), therefore addressing psychotic and behavioral manifestations. By acting on both transporters, SOM3355 modulates the dopaminergic tone in pre-cortical, cortical and mid-brain nuclei and limits the relative chronic hypodopaminergic tone in the basal ganglia, supporting the regulation of postsynaptic compensatory responses driven by excess cytosolic dopamine in presynaptic terminals. This might contribute to re-establishing cortico-striatal balance and potentially limiting the symptoms of HD beyond motor symptoms only, and therefore addressing behavioral, cognitive and psychiatric symptoms as well.

In comparative *in vitro* CNS target profiling assays of SOM3355 versus TBZ and α-DHTBZ, SOM3355 showed a clean, favorable binding signature that lacks the direct post-synaptic dopaminergic blockade characteristic of TBZ and its derivatives, further substantiating the clinical absence of pro-dyskinetic effects, which are instead observed in classical VMAT2 inhibitors. Of note, SOM3355 displayed affinity for serotonin receptors, while no activity appeared at the serotonin transporter, therefore potentially suggesting a partial, direct modulatory role on the serotonergic system. This may further support the synergistic psychiatric benefits observed in clinical studies, as 5-HT partial agonism is reportedly associated with anxiolytic and mood-stabilizing effects such as those exerted by compounds like buspirone (Loane and Politis, 2012; Cepeda et al., 2014).

### 3.1 Dopamine and β-adrenergic systems interactions

Dopamine and β-adrenergic systems interact in the brain in complex ways, influencing various functions like mood, motivation and cognitive processes (Lei, 2014; Xing et al., 2016; Creed et al., 2014). These systems do not operate in isolation; they have overlapping target regions and can directly or indirectly influence each other. Evidence is provided by i) colocalization of dopamine D_2_-like receptors and β-adrenergic receptors in neurons in several brain regions (Xing et al., 2016; Lee et al., 2020), suggesting they might form functional signaling complexes (Rebois et al., 2012), ii) modulation of signaling pathways with β-adrenergic receptors and dopamine D_1_-like receptors, both stimulating the production of cAMP (Undieh, 2010; Meitzen et al., 2011), while D_2_-like receptors can influence signaling pathways involving inhibitory G-proteins (Bibb, 2005), iii) direct and indirect interactions through dopamine that can indirectly affect β-adrenergic signaling through its influence on norepinephrine release and reuptake (Ranjbar-Slamloo and Fazlali, 2020; Xing et al., 2016).

The interplay between these systems is crucial for various brain functions. For example, they both play a role in the prefrontal cortex, where they modulate working memory and attention (Clark and Noudoost, 2014; Xing et al., 2016). In other areas, like the nucleus accumbens, dopamine and β-adrenergic signaling can influence reward and motivation (Aono et al., 2007; Xu et al., 2024). Acting only on dopamine or only on β-adrenergic systems may cause aberrant interactions between both systems and may contribute to neurological and psychiatric symptoms.

In essence, in HD the symptoms are not solely due to the dysfunction of one neurotransmitter in a single nucleus, *i.e*., of dopamine in the striatum. On the contrary, they arise from a complex interplay of neurotransmitter pathways, particularly within the cortico-striatal circuit, leading to the diverse motor, cognitive and psychiatric symptoms observed in HD patients. Moreover, dopamine levels may initially be elevated, potentially contributing to early symptoms like chorea, and later decline as the disease progresses, causing parkinsonism. Dysregulation of dopamine-glutamate interactions is also a key factor in the cortico-striatal pathway’s dysfunction (Rangel-Barajas and Rebec, 2016; Bunner and Rebec, 2016). The cortico-striatal pathway, which connects the cortex and striatum, is significantly affected in HD. This pathway relies on the coordinated activity of multiple neurotransmitters, and its dysfunction is central to the motor, cognitive and psychiatric symptoms observed in HD. SOM3355 may offer additional symptoms control beyond chorea through the broader pharmacologically targeted profile modulating dopaminergic activity in cortical and midbrain regions, alongside β-adrenergic tone in prefrontal areas. In line with these findings, the pharmacological properties of SOM3355 as a β-adrenergic antagonist support a broader spectrum of potential therapeutic effects. β-blockers are associated with improvements in certain cognitive performance, particularly in tasks involving memory and perceptual-motor coordination, possibly due to their anxiolytic effects (Bodner et al., 2012; Palac et al., 1990). Furthermore, evidence from other neuropsychiatric conditions indicates that β-blockers may reduce aggression (Ratey et al., 1987; Nevels et al., 2010; Luauté et al., 2016; Plantier et al., 2016), a symptom that can co-occur with cognitive and behavioral disturbances in HD. Based on published data on lipophilic β-blockers like propranolol in neuropsychiatric conditions, additional symptomatic domains in which SOM3355 may offer benefits include akathisia and tremor (Thippaiah et al., 2025; Naguy et al., 2023; Zhou et al., 2022). While these effects require further clinical validation, they are consistent with the known CNS actions of β-blockers (Molero et al., 2023). Importantly, SOM3355 may also possess disease-modifying potential, as suggested by mechanistic insights, although this remains to be confirmed in longitudinal studies.

## 4 Conclusion

In conclusion, SOM3355 exhibits a distinct, polyvalent pharmacological profile that differentiates it from currently available symptomatic treatments for HD and tardive dyskinesia. By combining a flexible, substrate-dependent modulation of VMAT2 with VMAT1 inhibition and β_1_-adrenergic antagonism, SOM3355 provides a robust mechanistic rationale for its favorable clinical efficacy and safety profile. Specifically, this unique pharmacology mitigates the liability for depression, suicidality and akathisia–adverse events that frequently limit the use of standard dopamine-depleting agents like TBZ and its derivatives. Beyond the effective management of motor symptoms such as chorea, the target signature of SOM3355 offers the potential to concurrently address the complex behavioral, psychiatric and cognitive disturbances associated with HD. This multifaceted mechanism of action positions SOM3355 as a promising therapeutic candidate, capable of managing complex neurological symptomatology without exacerbating the underlying disease burden.

## 5 Materials and methods

### 5.1 VMAT assays

#### 5.1.1 VMAT2 functional uptake assays in rat cortical vesicles

Screening and half-maximal inhibitory concentration (IC_50_) determination were performed at PerkinElmer. An *in vitro* VMAT2-mediated functional uptake assay was employed to rapidly evaluate potential VMAT2 inhibitors, following the procedure described by Sandoval et al. (2002). Briefly, rat brain cortical synaptic vesicles were purified by differential centrifugation, resuspended in assay buffer, and preincubated for 30 min at room temperature with either the positive reference control (reserpine), the comparative sample (TBZ) or individual test compounds from the screening set.

After preincubation, uptake was initiated by the addition of tritiated-dopamine ([^3^H]-DA) and the vesicle suspensions were incubated for 15 min at room temperature. The reaction was then terminated by vacuum filtration, and the radioactivity trapped on the filters was quantified by liquid scintillation spectrophotometry. For each test compound, the amount of radioactivity retained was compared with vehicle treated vesicles and expressed as percent inhibition relative to reserpine.

Following an initial screening at a single concentration of 10 µM in duplicate (n = 2), the IC_50_ of SOM3355 was determined by testing six concentrations ranging from 0.1 nM to 10 µM.

#### 5.1.2 VMAT2 rat vesicular monoamine transporter binding antagonist radioligand assay

[^3^H]-α-dihydrotetrabenazine ([^3^H]-α-DHTBZ), the active metabolite of TBZ, binding displacement assays were performed at Eurofins on rat brain membrane preparations (minus cerebellum). Assays were conducted with 1.0% dimethyl sulfoxide as vehicle and an incubation time of 30 min at 25 °C. The incubation buffer consisted of 25 mM 4-(2-hydroxyethyl)-1-piperazineethanesulfonic acid (HEPES), 100 mM potassium tartrate, 5 mM MgSO_4_, 0.1 mM ethylenediaminetetraacetic acid (EDTA), and 0.05 mM ethylene glycol-bis(β-aminoethyl ether)-N,N,N′,N′-tetraacetic acid (EGTA) at pH 7.4. Binding was measured with 10 nM [^3^H]-DHTBZ as ligand; nonspecific binding was defined in the presence of 10 µM Ro4-1284. Under these conditions, specific binding accounted for 80% of total signal, and the dissociation constant for the radioligand was 14.0 nM with a maximum number of binding sites of 1.60 pmol/mg protein. Data were quantified by radioligand binding, and significance was defined as ≥ 50% of maximal stimulation or inhibition.

#### 5.1.3 Comparative VMAT2 functional screening of β-blockers for dopamine uptake in rat striatal vesicles

An *in vitro* VMAT2 functional uptake assay was performed at the Medical University of Vienna, Center for Brain Research (Prof. Christian Pifl) to evaluate the VMAT2 inhibitory activity of SOM3355 and 22 β-blockers. DA uptake was measured in synaptic vesicles from rat striatum. Vesicle preparations were incubated in potassium phosphate buffer for 5 min at 30 °C with 0.1 µM [^3^H]-DA in the absence (vehicle control) or presence of reserpine and test compounds at varying concentrations. Nonspecific uptake was determined in the presence of 1 µM reserpine. Following incubation, membranes were filtered and washed, and filters were counted to quantify [^3^H]-DA retained in vesicles.

#### 5.1.4 Human VMAT2 functional uptake of neurotransmitters and binding

All human VMAT2 experiments were conducted at WuXi AppTec (Shanghai, China) using transiently transfected cell monolayers and vesicles prepared from a stable VMAT2-expressing HEK293 line. Radioligands were [^3^H]-reserpine (ViTrax, VT249), [^3^H]-α-DHTBZ (American Radiolabeled Chemicals, ART2119), [^3^H]-DA (PerkinElmer, NET673001MC), and [^3^H]-5-HT (PerkinElmer, NET498001MC). Radioactivity was detected on a MicroBeta^2^ plate reader after filtration on Unifilter-96 GF/C plates using a FilterMate harvester (PerkinElmer); MicroScint O or MicroScint 20 cocktails were used as specified below.

For cell-based binding, HEK293 or CHO-K1 cells were seeded at 2.0 × 10^4^ cells/well (96-well plates) and transiently transfected with human VMAT2 (NM_003054.6) cloned in pIRES2-EGFP (80 ng DNA/well; FuGENE® HD). Twenty-four hours after transfection, monolayers were washed with assay buffer (25 mM HEPES, 100 mM potassium tartrate, 5 mM MgSO_4_, 0.1 mM EDTA, 0.05 mM EGTA; pH 7.4) and permeabilized with digitonin (10 μg/mL, 10 min, 37 °C). [^3^H]-α-DHTBZ binding was performed at 6 nM radioligand for 60 min at 37 °C; non-specific binding (NSB) was defined with 10 µM tetrabenazine (TBZ). [^3^H]-reserpine binding used an ATP-containing buffer (25 mM HEPES, 100 mM potassium tartrate, 5 mM glucose, 4 mM KCl, 4 mM MgSO_4_, 0.1 mM EDTA, 0.05 mM EGTA, 4 mM ATP, 0.5% BSA; pH 7.4) with NSB defined by 10 µM reserpine. After incubation, monolayers were washed cold, lysed with 10% NaOH, mixed with MicroScint O, and counted.

For vesicle studies, a G418-selected VMAT2-HEK293 stable line was generated (HEK293 selection at 600 μg/mL), verified by [^3^H]-α-DHTBZ binding, and expanded for membrane preparation. Vesicles were prepared by homogenization in 0.32 M sucrose, differential centrifugation (2,000 g/10 min; 10,000 g/30 min), osmotic shock and restoration, followed by clarification (20,000 g/20 min) and ultracentrifugation (87,000 g/60 min). Pellets were resuspended in assay buffer, quantified by BCA, aliquoted, and stored at −80 °C.

Vesicle binding to [^3^H]-α-DHTBZ used 1.5 µg vesicular protein per well in 25 mM HEPES, 100 mM potassium tartrate, 5 mM MgSO_4_ (pH 7.4) with 4.5 nM radioligand for 60 min at room temperature; NSB was defined with 10 µM TBZ. [^3^H]-reserpine binding was carried out at 2 nM radioligand with 50 µg vesicles per well in 25 mM HEPES, 100 mM potassium tartrate, 4 mM KCl, 2 mM MgSO_4_, 0.1 mM EDTA, 0.05 mM EGTA, 1 µM pargyline, 3 mM ATP (pH 7.4) supplemented with 0.5% BSA, incubated 60 min at 30 °C; NSB was defined with 10 µM reserpine. Reactions were terminated by rapid filtration on GF/C plates pre-soaked with 0.3% PEI ([^3^H]-α-DHTBZ) or 0.5% BSA ([^3^H]-reserpine), washed 6x with cold buffer, dried, and read with MicroScint 20 on MicroBeta^2^.

Vesicle uptake of [^3^H]-DA and [^3^H]-5-HT employed an ATP-dependent buffer (25 mM HEPES, 100 mM potassium tartrate, 4 mM KCl, 2 mM MgSO_4_, 0.1 mM EDTA, 0.05 mM EGTA, 1 µM pargyline, 3 mM ATP; pH 7.4) containing 0.5% BSA. Protein loads were ∼10 µg/well for dopamine and ∼5 µg/well for serotonin. Substrate concentrations were 150 nM [^3^H]-DA (30 °C, 30 min) and 150 nM [^3^H]-5-HT (30 °C, 20 min). Uptake was terminated by filtration on BSA-soaked GF/C plates, washed 6x cold, dried, and counted with MicroScint 20. TBZ (10 µM) and excess unlabeled substrate were included as controls; blank vesicles from non-transfected HEK293 were run in parallel. Saturation/kinetic series (2.5–500 nM) were used to determine Kd/Km where applicable.

#### 5.1.5 VMAT2 stabilization assessment by DSF

Expression of rat VMAT2 in insect cells, protein purification and DSF assessment were conducted at the University of Bergen (Prof. Aurora Martinez’ Lab). Recombinant rat VMAT2-His was expressed in Sf9 insect cells using the baculovirus expression system. Cells were grown at 28 °C to a density of 2.5 × 10^6^ cells/ml and infected with baculovirus containing the rat VMAT2-his ORF in its genome. Cell infection was monitored by assessing YFP fluorescence from a reporter gene inserted in the baculovirus genome. Cells were harvested 3 days after growth arrest at 6000 g in a Beckman Avanti J-2 centrifuge. Harvested cells were lysed by sonication and the insoluble fraction containing membranes were pelleted by ultracentrifugation at 180,000 g for 1 h in a Beckman Coulter Optima MAX-TL ultracentrifuge. Membranes were resuspended in resuspension buffer (50 mM HEPES, pH 7.5, 150 mM NaCl and complete protease inhibitors at a wet weight concentration of 250 μg/mL). Membranes were washed in a high salt resuspension buffer (50 mM HEPES, pH 7.5, 500 mM NaCl and complete protease inhibitors (Roche)), and resuspended and homogenized in resuspension buffer containing 10% glycerol before storage at −80 °C. For protein purification the membranes were thawed and diluted to a protein concentration of approximately 5 mg/mL and solubilized in resuspension buffer containing 1% n-Dodecyl-β-D-maltoside (DDM) and 0.2% cholesterol hemisuccinate (CHS) for 1.5 h on a rotary wheel at 4 °C. Unsolubilized material was removed by ultracentrifugation at 100,000 g for 30 min, and the supernatant containing solubilized membranes was loaded onto a 5 mL HiTrap TALON crude column (Cytiva). The column was washed with >20 column volumes of wash buffer (50 mM HEPES, pH 7.5, 150 mM NaCl, 5 mM imidazole, 0.05% DDM, 0.01% CHS and complete protease inhibitors, before being eluted on an akta Pure protein purification system in elution buffer (50 mM HEPES, pH 7.5, 150 mM NaCl, 250 mM imidazole, 0.05% DDM, 0.01% CHS and complete protease inhibitors). Elution fractions containing rat VMAT2 were concentrated in an Amicon Ultra 100 kDa spin column and loaded on a Superdex 200 increase 10/300 size exclusion chromatography column. The eluted fractions were evaluated by SDS-PAGE, and the fractions containing monomeric, purified VMAT2 were collected and used for further analysis. Purified rat VMAT2-His at a final concentration of 50 ng/μL was mixed with the thiol-sensing dye diethylamino-3-(4-maleimidophenyl)-4-methylcoumarin (CPM) and increasing concentrations of SOM3355 (the racemic mixture), TBZ or DMSO control in triplicates and preincubated at 20 min at room temperature before DSF analysis. DSF was performed with 10 µL reaction volumes in 384 well plates, and the rat VMAT2 protein melting temperature was determined on a Light Cycler 480 ii RT-PCR system from Roche. The sample was heated with a temperature ramp of 2 °C/min, from 25 °C to 95 °C. Data analysis was performed using HTSDSF Explorer that automatically determines the protein midpoint melting temperature (T_m_) based on the second derivative of the melting curves.

#### 5.1.6 VMAT1-mediated functional uptake of serotonin

VMAT1 functional assays were performed at the Medical University of Vienna, Center for Brain Research (Prof. Christian Pifl) using CHO cells stably transfected with rat VMAT1 (pcDM8) together with a geneticin resistance plasmid (pRcCMV) introduced by the calcium phosphate method; clones were selected in geneticin and subsequently in MPP^+^ to enrich transporter activity prior to membrane preparation. Vesicular membrane fractions were generated from confluent cultures by resuspension in 0.3 M sucrose/25 mM Tris HCl (pH 7.4) containing pargyline and protease inhibitors, followed by controlled sonication and clarification (16,100 g, 10 min, 4 °C); the resulting supernatant was used immediately for uptake experiments to preserve transporter function.

Initial rates of [^3^H]-5-HT uptake were measured in a 0.13 M potassium phosphate buffer (pH 7.4) containing MgATP and pargyline/ascorbic acid to maintain substrate stability. Test compounds were prepared in DMSO as concentrated stocks and diluted into assay buffer; in triplicate tubes, buffer or 1 µM reserpine (positive control) was mixed with test article, 60 mM MgATP, and 3 µM 5-HT spiked with 0.1 µCi [^3^H]-5-HT (PerkinElmer NET1167250UC). Uptake was initiated by adding the freshly prepared VMAT1 vesicles (∼150 µL membrane fraction) at 30 °C for 5 min in a total volume of 1.5 mL. Reactions were terminated with 2.5 mL ice cold buffer and collected by vacuum filtration onto GF/B filters pre-soaked in 1% polyethylenimine (Brandel harvester); filters were washed twice with 3 mL cold buffer, transferred to scintillation vials, incubated with cocktail at 50 °C (2 h), and counted after vigorous mixing.

### 5.2 CNS target binding panel

CNS target profiling was performed at Eurofins (Celle l’Evescault, France) using a standardized panel of 35 receptors, ion channels, transporters and enzymes. Each compound was tested at a single concentration of 10 µM in duplicate (n = 2). For binding assays, results were expressed as percent inhibition of control specific binding; for enzyme assays, as percent inhibition of control enzyme activity.

#### 5.2.1 Radioligand binding assays

Binding was performed on human recombinant preparations (CHO or HEK293 host cells) or tissue membranes, with scintillation counting of bound radioactivity. Target specific radioligands were used as follows: α_1A_ adrenergic receptor ([^3^H]-prazosin); α_2B_/α_2C_ adrenergic receptors ([^3^H]-RX 821002); D_1_ dopamine receptor ([^3^H]-SCH 23390); D_2s_ dopamine receptor ([^3^H]-7-OH-DPAT (agonist radioligand)); D_2L_, D_3_, D_4.4_ dopamine receptors ([^3^H]-methylspiperone); 5-HT_1A_ serotonin receptor ([^3^H]-8-OH-DPAT (agonist radioligand)); 5-HT_2A_/5-HT_2C_ serotonin receptors(^125^I-DOI); 5-HT_3_ serotonin receptor ([^3^H]-BRL-43694); H_1_ histamine receptor ([^3^H]-pyrilamine); CB_1_ cannabinoid receptor ([^3^H]-CP-55940 (agonist radioligand)); sigma (non-selective) site ([^3^H]-DTG); androgen receptor ([^3^H]-methyltrienolone). Ion channel and transporter assays used [^3^H]-flunitrazepam (central benzodiazepine site), [^3^H]-CGP-39653 (N-methyl-D-aspartate receptor), [^3^H]-tenocyclidine (phencyclidine binding site), [^125^I]-apamin (SK Ca^2+^ channel), [^3^H]-batrachotoxin (Na^+^ channel site 2), [^35^S]-tert-butyl bicyclophosphorothionate [gamma-aminobutyric acid (GABA) gated Cl^−^ channel], [^3^H]-nisoxetine (NET), [^3^H]-benocyclidine (DAT), [^3^H]-GABA (GABA transporter; in the presence of isoguvacine and baclofen), and [^3^H]-imipramine (5-HT transporter). Additional binding targets included [^125^I]-tumor necrosis factor α (tumor necrosis factor α) and [^125^I]-2-iodomelatonin (melatonin receptor subtype MT3/ML2). Non-specific binding was defined with the appropriate competitor for each assay (e.g., epinephrine, butaclamol, (±)-DOI, MDL-72222, picrotoxinin, desipramine, benocyclidine, GABA, imipramine), and incubations were carried out for 60–120 min at room temperature, 37 °C, or 4 °C as required by the target. A threshold of ≥50% inhibition was used to define significant activity. Effects between 25% and 50% were classified as weak to moderate, while values below 25% were considered non-significant and attributable to variability of the signal around the control level. Low to moderate negative values were considered as background noise.

#### 5.2.2 IC_50_ determination at dopamine and norepinephrine transporters

Eurofins performed two functional radiolabeled uptake assays to study the inhibitory activity at the dopamine transporter (DAT) and the norepinephrine transporter (NET) respectively. For each transporter, five concentrations of the test compounds (1 nM, 10 nM, 100 nM, 1 μM, 10 µM) were evaluated in duplicate (n = 2) to generate concentration-response data and derive IC_50_ values.

For the DAT assay, human recombinant CHO-S cells were incubated with the test compounds, and uptake was quantified by measuring [^3^H]-DA accumulation following a 10 min incubation. Nomifensine (10 µM) served as the control inhibitor (reference IC_50_ = 0.017 µM). The IC_50_ was defined as the concentration producing 50% inhibition of [^3^H]-DA uptake relative to the response obtained with the nomifensine control.

For the NET assay, human recombinant Madin-Darby canine kidney cells were used under analogous conditions, with uptake quantified by [^3^H]-norepinephrine accumulation after a 15 min incubation. Desipramine (10 µM) served as the control inhibitor (reference IC_50_ = 1.9 nM). The IC_50_ was defined as the concentration producing 50% inhibition of [^3^H]-norepinephrine uptake relative to the desipramine control. A threshold of ≥50% inhibition was used to define significant activity.

#### 5.2.3 Selected enzyme inhibition assays

Acetylcholinesterase activity (human recombinant, HEK293) was measured using acetylthiocholine (400 µM) with photometric detection of the Ellman chromophore (30 min, room temperature). GABA transaminase activity (rat brain) was quantified fluorometrically with GABA (9 mM) and α-ketoglutarate (9 mM) (60 min, 37 °C). Tyrosine hydroxylase activity (rat striatum) was determined by scintillation counting of [^3^H]-tyrosine (10 µM) conversion to [^3^H]-H_2_O (40 min, 37 °C). A threshold of ≥50% inhibition was used to define significant activity. Effects between 25% and 50% were classified as weak to moderate, while values below 25% were considered non-significant and attributable to variability of the signal around the control level. Low to moderate negative values were considered as background noise.

### 5.3 Adrenergic pharmacology characterization

#### 5.3.1 *In vitro* profiling of α/β-adrenergic antagonism

*In vitro* assays for adrenergic activity were performed by Eurofins on human α_1A_, α_2A_, β_1_ and β_2_ adrenoceptors transiently expressed in selected host cells (α_1A_ in CHO, α_2A_ in rat basophilic leukemia cells, β_1_ in HEK293, β_2_ in CHO). Antagonist responses were quantified against fixed reference agonists: epinephrine 3 nM for α_1A_/α_2A_ at room temperature with real-time intracellular Ca^2+^ fluorimetry, and isoproterenol 3 nM (β_1_) or 10 nM (β_2_) with cAMP-homogeneous time-resolved fluorescence after 30 min incubation at room temperature. Concentration-response series for test compounds were run in replicate wells; IC_50_ values were obtained by non-linear regression of mean replicate data using a Hill-equation. Responses were expressed as % inhibition of the control agonist response (antagonism) or % of control (agonism) under the same stimulus and detection conditions.

#### 5.3.2 Adrenergic assessments in animal models

Adrenergic pharmacology characterization of bevantolol (SOM3355) in animal models was historically conducted by Warner-Lambert Company, LLC, and Nippon Chemiphar Co., Ltd.

All animal housing, care, and experimental protocols complied with the institutional guidelines and internationally recognized standards for laboratory animal welfare applicable at the time the studies were performed (1974–1980). The inclusion of these historically validated *in vivo* and *ex vivo* data aligns with the 3Rs ethical framework (specifically, the principle of Reduction), avoiding the unnecessary duplication of animal experiments and the unjustified sacrifice of new animals (including higher species such as dogs) to replicate established pharmacological profiles.

##### 5.3.2.1 Rabbit thoracic aorta model of α_1_-antagonism

Male Japanese white rabbits (2–3 kg, Shin Nippon Doubutsu) were euthanized by exsanguination; the thoracic aorta was excised immediately and the endothelium removed. Spiral strips were prepared and mounted with a 1.0 g preload in a 20 mL organ bath containing Krebs solution (NaCl 118 mM, KCl 4.5 mM, CaCl_2_ 2.5 mM, MgSO_4_ 1.0 mM, KH_2_PO_4_ 1.0 mM, NaHCO_3_ 25 mM, glucose 6.0 mM), continuously aerated with 95% O_2_/5% CO_2_ at 37 °C. Preparations equilibrated for 1 h before cumulative norepinephrine (10^−9^-3 × 10^-5^ M) was applied to generate a stable control concentration–response curve; baths were then rinsed and tissues re-equilibrated. Test compounds articles were introduced as a 30 min pre-incubation at a fixed concentration (10^−5^ M), followed by a second cumulative norepinephrine series under identical conditions. Contractions were recorded isotonically, and α_1_-antagonism was quantified from rightward shifts of the norepinephrine curve; pA_2_ values were estimated using standard cumulative dose-response methodology, using the Van Rossum calculation tables.

##### 5.3.2.2 *In vivo* β_1_-versus α_1_-selectivity in dogs

Adult cross-breed dogs (∼10 kg) were anesthetized using intravenous injections of pentobarbital sodium (Siegfried Zofingen) at 10–15 mg/kg. Anesthesia was maintained through subcutaneous injections of urethane and α-chloralose at 500 mg/kg and 40 mg/kg, respectively. Tracheotomy was performed using a cannula to secure airways. We measured the systemic blood pressure with an electro-manometer (AP-621G, NIHON KOHDEN CORPORATION) via a pressure transducer (TP-101T, NIHON KOHDEN CORPORATION) through the polyethylene cannula that was inserted into a femoral artery, and then measured the heart rate by synchronizing a heart rate monitor (AT-601G, NIHON KOHDEN CORPORATION). After physiological stabilization, cumulative intravenous phenylephrine (α_1_ stimulant; 2–20 μg/kg) and isoproterenol (β_1_ stimulant; 0.02–0.5 μg/kg) were given in ascending order to generate baseline dose-response curves for the vasopressor and positive chronotropic reactions, respectively. The investigational article was then administered intraduodenally at 10 mg/kg or 30 mg/kg; 30 min post-dose, cumulative dose-response series were repeated (phenylephrine 5–50 μg/kg; isoproterenol 0.5–10 μg/kg) under identical recording conditions. In a complementary preparation, a right-ventricular strain-gauge and right cardioaccelerans nerve (decentralized) were used to quantify β_1_ cardiac blockade: dogs received cumulative intravenous test-compound doses (0.01–10 mg/kg, half-log increments, 20-min intervals), with isoproterenol (0.3 μg/kg intravenous) and nerve-stimulation (3 Hz, 2x threshold) challenges interposed between dose steps; a catecholamine-depleted cohort was evaluated after reserpine pretreatment. For each animal, parallelism of pre/post curves was verified and DR with 95% CIs were obtained using a parallel-line assay (2 × 3-point); the β_1_/α_1_-selectivity was expressed as DR_ISO/DR_PE at doses showing significant rightward shifts (multiple comparisons by Tukey’s test). In the 30 mg/kg intraduodenal group, plasma samples were collected at 30 min for exposure-effect correlation with the phenylephrine DR.

##### 5.3.2.3 Guinea-pig right atrium β_1_-antagonism

Male Hartley guinea pigs (240–570 g) were euthanized by exsanguination. Immediately after death, the right atrium was removed and kept at 37 °C and suspended by applying a load of 0.5 g in 10 mL of organ bath filled with a Krebs-Henseleit solution (composition: NaCl 118 mM, KCl 4.7 mM, CaC1_2_ 2.5 mM, MgSO_4_ 1.0 mM, NaHCO_3_ 25.0 mM, KH_2_PO_4_ 1.2 mM, glucose 10.0 mM) that was aerated with mixed gas (95% O_2_ + 5% CO_2_). Contractile force and sinus rhythm were measured by an amplifier for strain pressure (AP-621G, Nihon Kohden) and an instantaneous heart rate meter unit (AT600G, Nihon Kohden) via a FD pickup (TB 612T, Nihon Kohden), and the results were recorded on a heat recorder (WT-687G, Nihon Kohden). Contractile force and sinus rate were recorded continuously with standard mechanical/chronotropic transducers. After establishing stable cumulative isoproterenol concentration-response curves (chronotropic and inotropic endpoints), tissues were pre-incubated for 5 min with the test compounds, followed by a second cumulative isoproterenol series under identical conditions to quantify competitive β_1_ blockade (isoproterenol cumulative concentrations ranged from 10^−10^–10^−7^ M, and β_1_-antagonist concentrations were applied cumulatively at 10^−8^, 3 × 10^−8^, and 10^−7^ M). Antagonism was analyzed by Schild regression and pA_2_ values were computed using the Tallarida statistical method.

## Data Availability

The raw data supporting the conclusions of this article will be made available by the authors, without undue reservation.
