# Supplementary material for: SOM3355: a unique pharmacological profile combining VMAT1 inhibition, VMAT2-mediated dopamine modulation, and β1-adrenergic antagonism for the treatment of movement and neuropsychiatric disorders
Source: Front Pharmacol. 2026 May 19;17:1824708. doi: 10.3389/fphar.2026.1824708 (PMC13226201; doi:10.3389/fphar.2026.1824708)
Supplement: Supplementary file 1 [file Supplementaryfile1.docx]

Supplementary Material

# Supplementary Figures

**
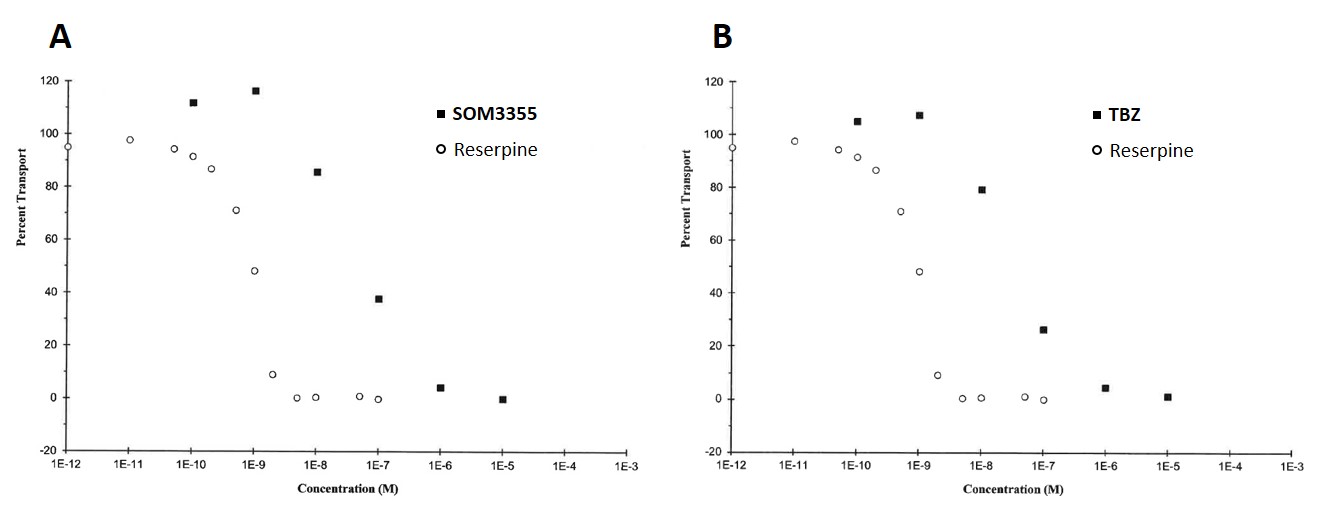
Supplementary Figure 1.** Dose-response curves of **(A)** SOM3355 and **(B)** TBZ for VMAT2-mediated [^3^H]-DA uptake into rat cortical vesicles.


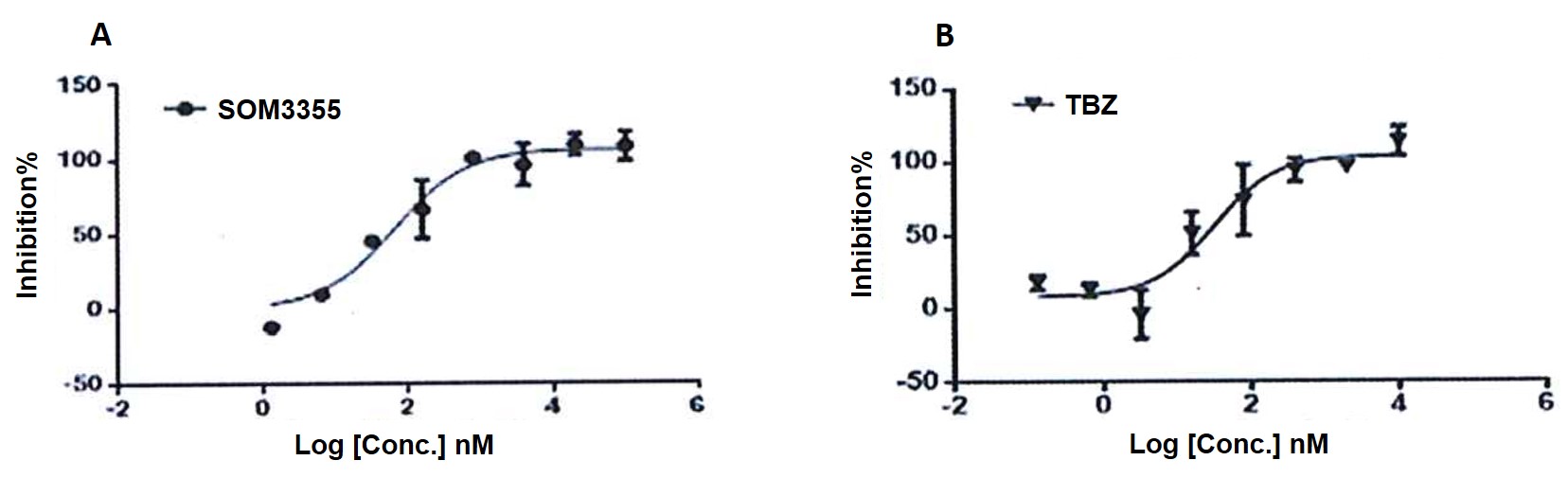


**Supplementary Figure 2.** Dose-response curves of **(A)** SOM3355 and **(B)** TBZ for VMAT2-mediated [^3^H]-DA uptake into human vesicles from transfected HEK293 cells.

**
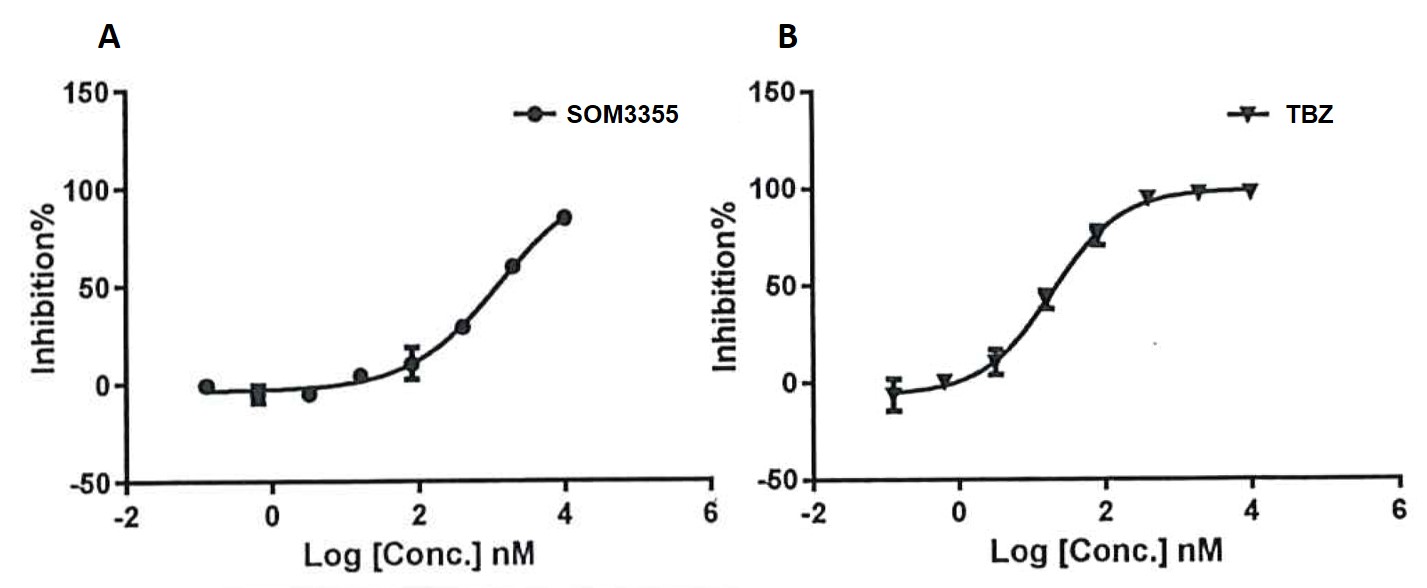
**

**Supplementary Figure 3.** Dose-response curves of **(A)** SOM3355 and **(B)** TBZ for VMAT2 [³H]-α-DHTBZ displacement in human VMAT2 transfected HEK293 cells.

**
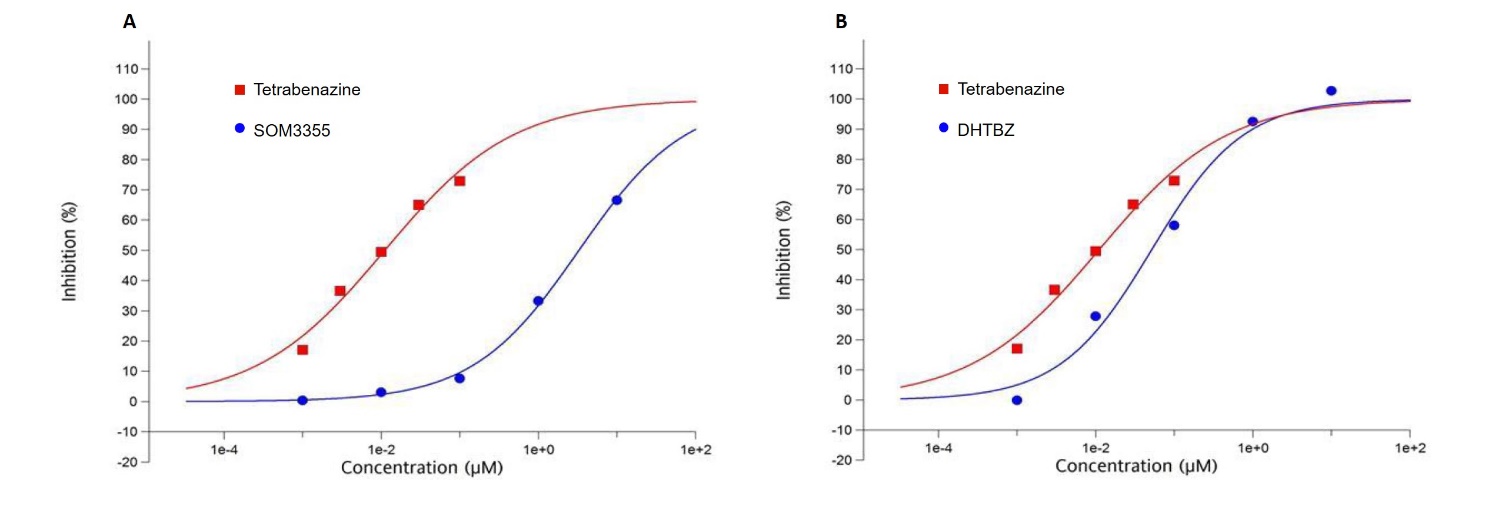
**

**Supplementary Figure 4.** Dose-response curves of **(A)** SOM3355 (Ki = 1.91 µM) and **(B)** α-DHTBZ (Ki = 0.030 µM) for VMAT2 [³H]-α-DHTBZ displacement in rat brain membranes.

**
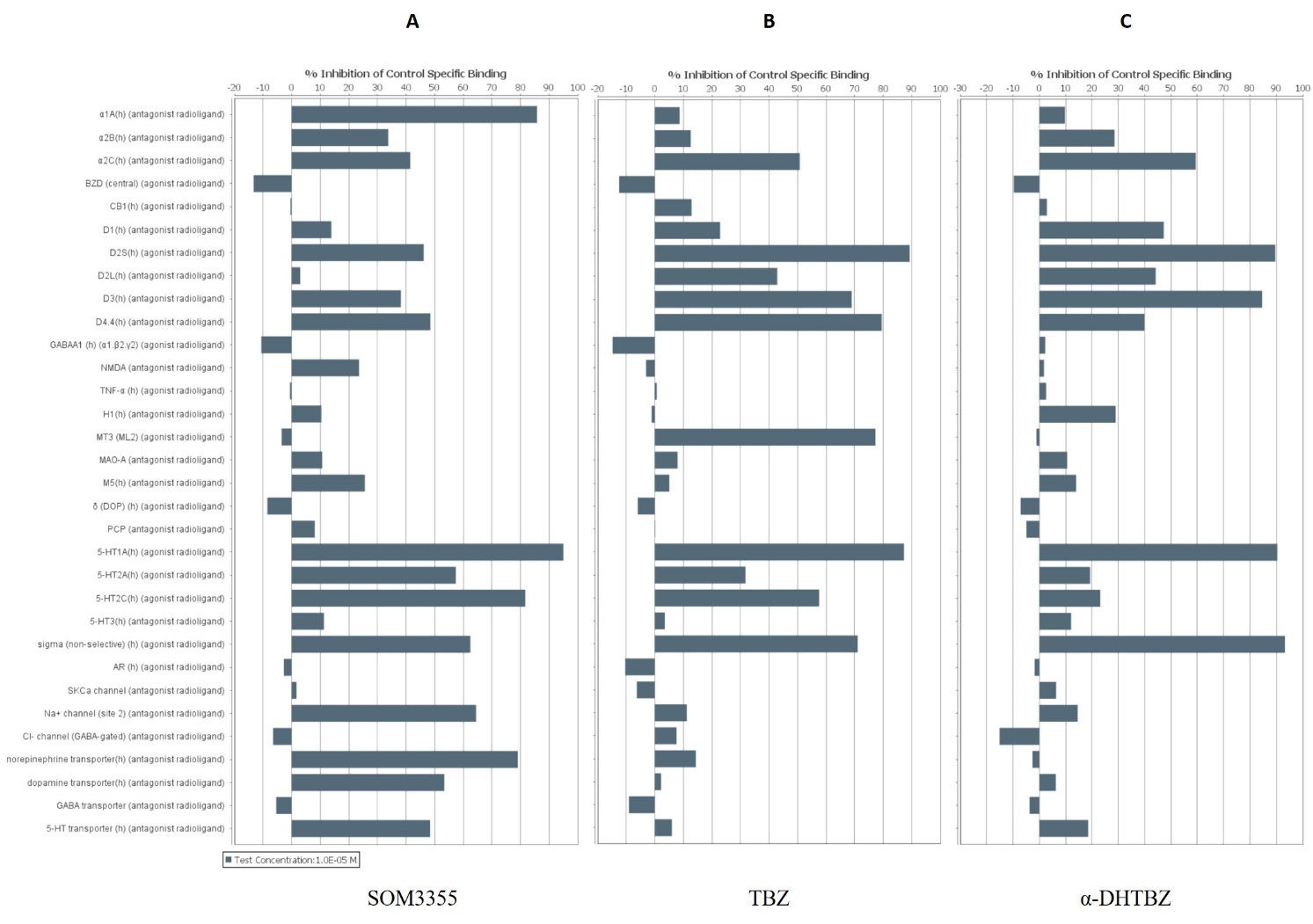
 Supplementary Figure 5.** Comparative CNS radioligand binding panel histograms for **(A)** SOM3355, **(B)** TBZ, and **(C)** α-DHTBZ, tested in duplicate at a single concentration of 10 µM.

**
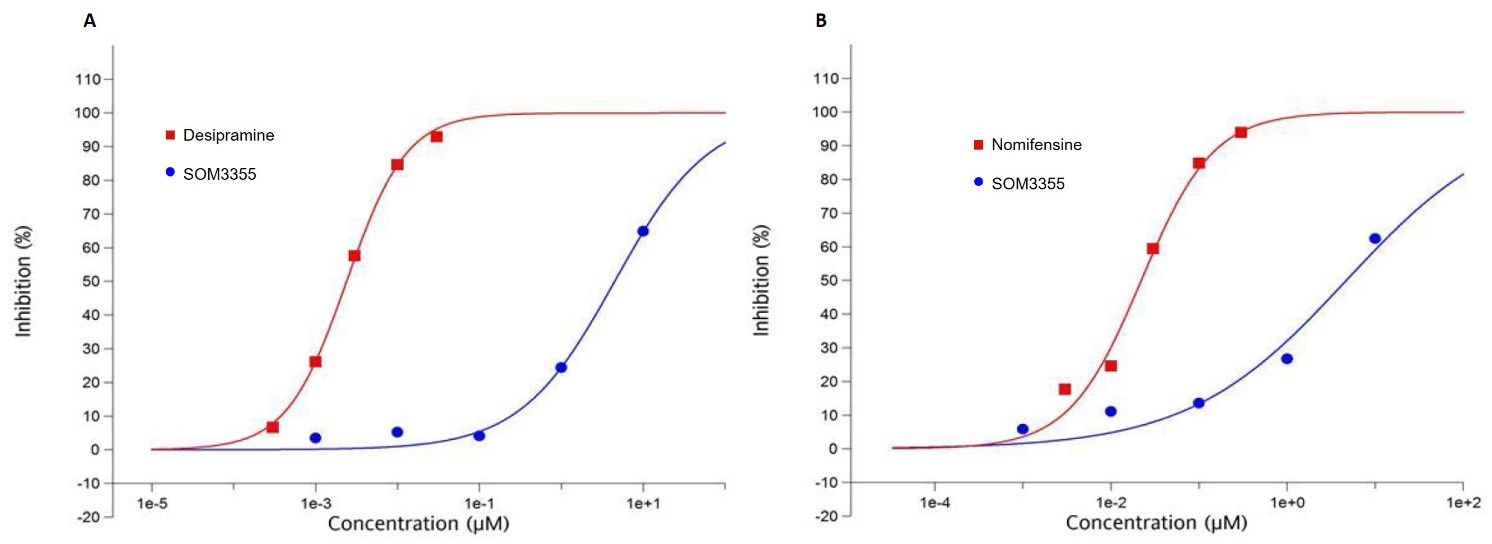
Supplementary Figure 6.** Dose-response curves of SOM3355 for **(A)** NET (IC_50_ = 4.47 μM) inhibition in MDCK cells and **(B)** DAT (IC_50_ = 4.68 μM) inhibition in CHO-S cells.

**
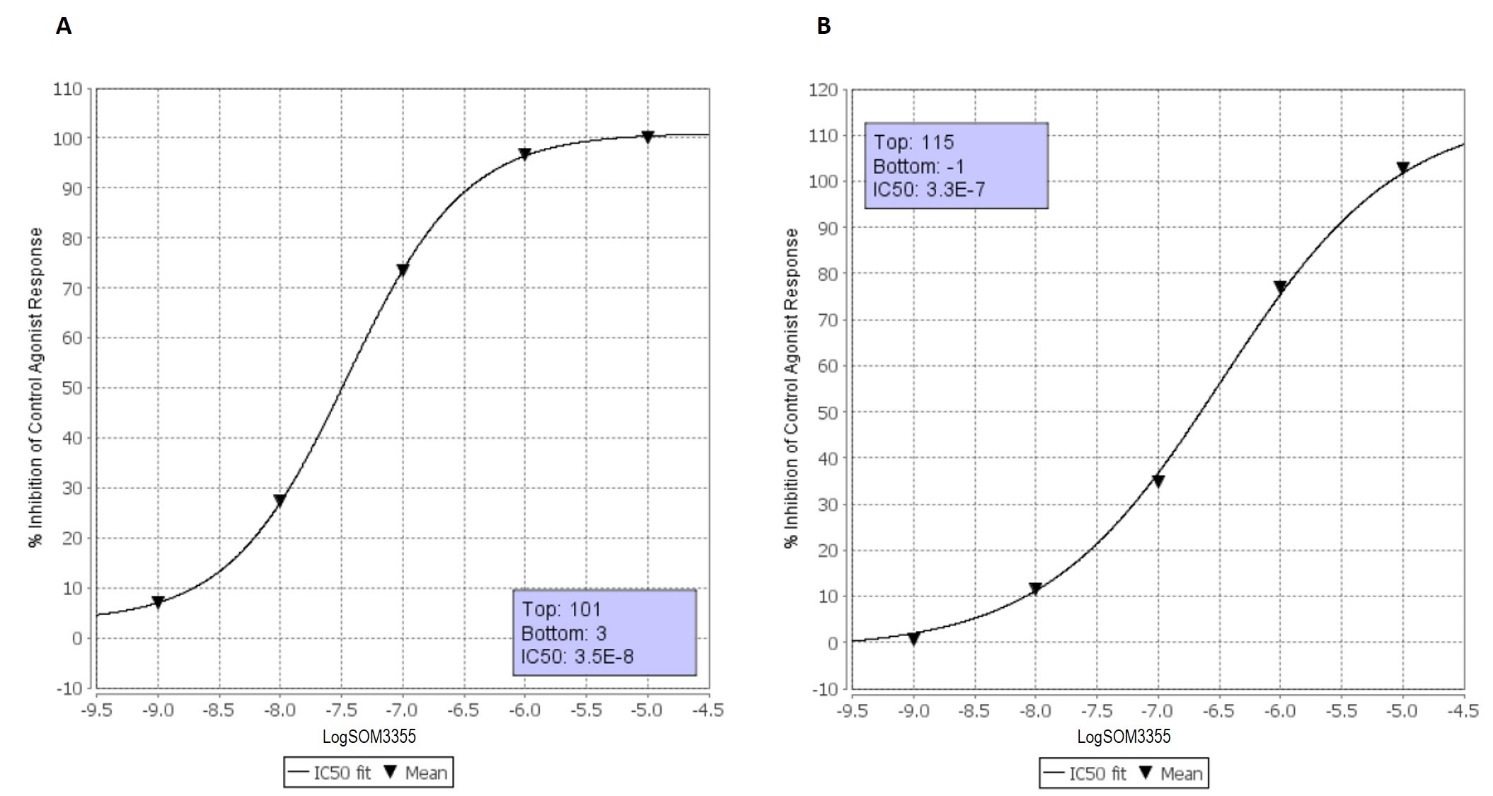
**

**Supplementary Figure 7.** Dose-response curve of SOM3355 tested for antagonist effect at (**A**) human β_1_-adrenergic receptor (HEK293 cells) and (**B**) β_2_-adrenergic receptor (CHO cells).


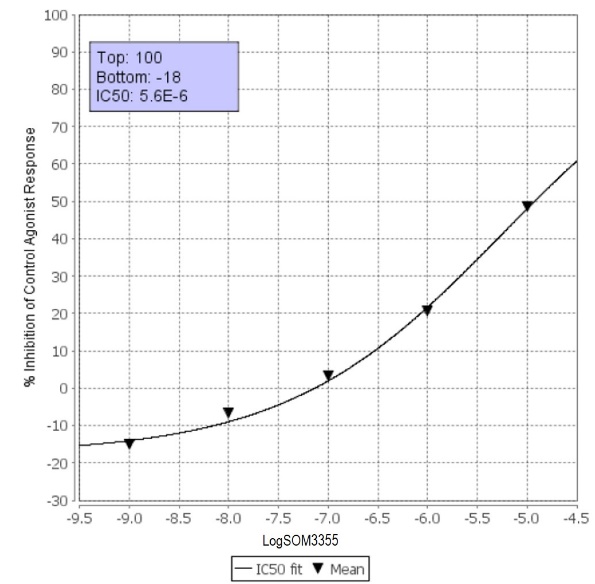


**Supplementary Figure 8.** Dose-response curve of SOM3355 tested for antagonist effect at human α_1A_ receptor (CHO cells).

# Supplementary Tables

**Supplementary Table 1.** Comparative screening of 23 β-blocking compounds tested in triplicates at a single concentration of 10 μM to inhibit initial rates of VMAT2-mediated [^3^H]-DA uptake into rat striatal synaptic vesicles. ND = Not Determined.

| **Compound Name** | **IC_50_ (μM)** |
| --- | --- |
| SOM3355 | 0.098 ± 0.006 |
| Propanolol HCl | 7.470± 1.380 |
| Bucindolol | 0.453 ± 0.101 |
| Carteolol HCl | ND (Inhibition < 50%) |
| Nadolol | ND (Inhibition < 50%) |
| Oxprenolol HCl | ND (Inhibition < 50%) |
| Acebutolol HCl | ND (Inhibition < 50%) |
| Atenolol | ND (Inhibition < 50%) |
| Betaxolol HCl | ND (Inhibition < 50%) |
| Bisoprolol hemifumarate | ND (Inhibition < 50%) |
| Celiprolol HCl | ND (Inhibition < 50%) |
| Esmolol HCl | ND (Inhibition < 50%) |
| Landiolol HCl | ND (Inhibition < 50%) |
| Butoxamine HCl | ND (Inhibition < 50%) |
| SR 59230A HCl | 3.900 ± 0.260 |
| Timolol maleate | 5.22 0 ± 0.570 |
| Sotalol HCl | ND (Inhibition < 50%) |
| Carvedilol | 0.227 ± 0.053 |
| Labetalol HCl | 0.202 ± 0.008 |
| Penbutolol | 6.820 ± 1.970 |
| Pindolol | ND (Inhibition < 50%) |
| Metoprolol tartrate | ND (Inhibition < 50%) |
| Nebivolol | 0.008 ± 0.0054 |

**Supplementary Table 2.** Comparative screening of SOM3355, TBZ, and trans-(2,3)-DHTBZ tested in triplicates at a single concentration of 10 μM to inhibit initial rates of VMAT2-mediated [^3^H]-5-HT uptake into rat VMAT1-expressing membrane vesicles from transfected CHO cells.

| **Compound Name** | **Uptake in presence of**  **10 μM compound ± SD**  **(% of control)** | **% Inhibition** |
| --- | --- | --- |
| SOM3355 | 24.1 ± 1.2 | -106 |
| TBZ | 102.0 ± 4.7 | 3 |
| trans-(2,3)-DHTBZ | 51.1 ± 2.5 | -65 |

**Supplementary Table 3.** Screening results for SOM3355, TBZ, and α-DHTBZ, tested at 10 μM in duplicates for inhibition of acetylcholinesterase (HEK293 cells), GABA transaminase (rat brain) and tyrosine hydroxylase (rat striatum).

| **Target** | **SOM3355** | **TBZ** | **α-DHTBZ** |
| --- | --- | --- | --- |
| **Acetylcholinesterase** | 13.8 | 8.8 | 14.8 |
| **GABA transaminase** | -8.0 | -4.3 | -8.4 |
| **Tyrosine hydroxylase** | -5.9 | -5.4 | -4.0 |
